# Primary thyroid-like low-grade nasopharyngeal papillary adenocarcinoma: A case report and literature review

**DOI:** 10.1097/MD.0000000000008851

**Published:** 2017-11-27

**Authors:** Wan-Lin Zhang, Shuang Ma, Lauren Havrilla, Lin Cai, Cheng-Qian Yu, Shuai Shen, Hong-Tao Xu, Liang Wang, Juan-Han Yu, Xu-Yong Lin, Endi Wang, Lian-He Yang

**Affiliations:** a Department of Pathology, First Affiliated Hospital and College of Basic Medical Sciences, China Medical University; b Department of Neurology, Sheng Jing Hospital of China Medical University, Shenyang, Liaoning, China; c Department of Pathology, Duke University Medical Center, Durham, NC.

**Keywords:** nasopharynx, papillary adenocarcinoma, thyroid, TTF-1

## Abstract

**Rationale:**

Primary thyroid-like low-grade nasopharyngeal papillary adenocarcinoma (TL-LGNPPA) is an extremely rare malignant nasopharyngeal tumor with features resembling papillary thyroid carcinoma including nuclear positive expression of thyroid transcription factor-1 (TTF-1).

**Patient concerns:**

A 64-year-old male presented with nasal bleeding and a foreign body sensation of the nasopharynx. Laryngoscopy revealed a 2.0-cm broad-based mass with a smooth surface on the posterior wall of the nasopharynx. A biopsy was obtained.

**Diagnoses:**

Histopathologic examination demonstrated tumor cells arranged in both papillary and glandular architecture. The tumor cells express nuclear immunoreactivity for TTF-1. The diagnosis of TL-LGNPPA was made.

**Interventions:**

After the patient was diagnosed with TL-LGNPPA, he underwent complete surgical resection.

**Outcomes:**

There was no recurrence or evidence of metastatic disease at the 12-month follow-up.

**Lessons:**

TL-LGNPPA is easy to misdiagnose as metastatic papillary thyroid carcinoma or other relative primary adenocarcinomas. It is important to have a broad differential diagnosis and know the key features of each entity because the prognosis and clinical treatment of each may differ.

## 1 Introduction

Primary nasopharyngeal adenocarcinoma can be mainly divided into 2 groups: those originating from the surface mucosal epithelium, such as low-grade nasopharyngeal papillary adenocarcinomas (LGNPPAs), and those which originate from the submucosal seromucous salivary glands,^[1]^ including low-grade adenocarcinoma, mucoepidermoid adenocarcinoma, and adenoid cystic carcinoma.^[2–4]^ In 1988, Wenig et al^[5]^ first described LGNPPAs as a distinct entity based on a series of nine patients with “conventional” adenocarcinomas in the nasopharyngeal region which had an indolent clinical behavior and low-grade histological features. Also, arising from the surface epithelium is the extremely rare but malignant primary thyroid-like low-grade nasopharyngeal papillary adenocarcinoma (TL-LGNPPA).^[1]^

Here we present a case of a 64-year-old Asian male with primary TL-LGNPPA. After an extensive literature review, we are able to summarize the key diagnostic features, clinical treatment, and prognosis of TL-LGNPPA.

## 2 Case report

A 64-year-old Asian male presented with nasal bleeding and a foreign body sensation within the nasopharynx. He never experienced these symptoms before. Physical examination was performed, but no obvious abnormality was found. Laryngoscopy revealed a 2.0-cm broad-based mass with a smooth surface located on the posterior wall of the nasopharynx. Subsequent imaging studies including chest and abdominal computed tomography (CT) scans, as well as a thyroid ultrasound, did not reveal any abnormalities. The patient underwent an incisional biopsy and subsequently completed surgical resection.

**Figure 1:**
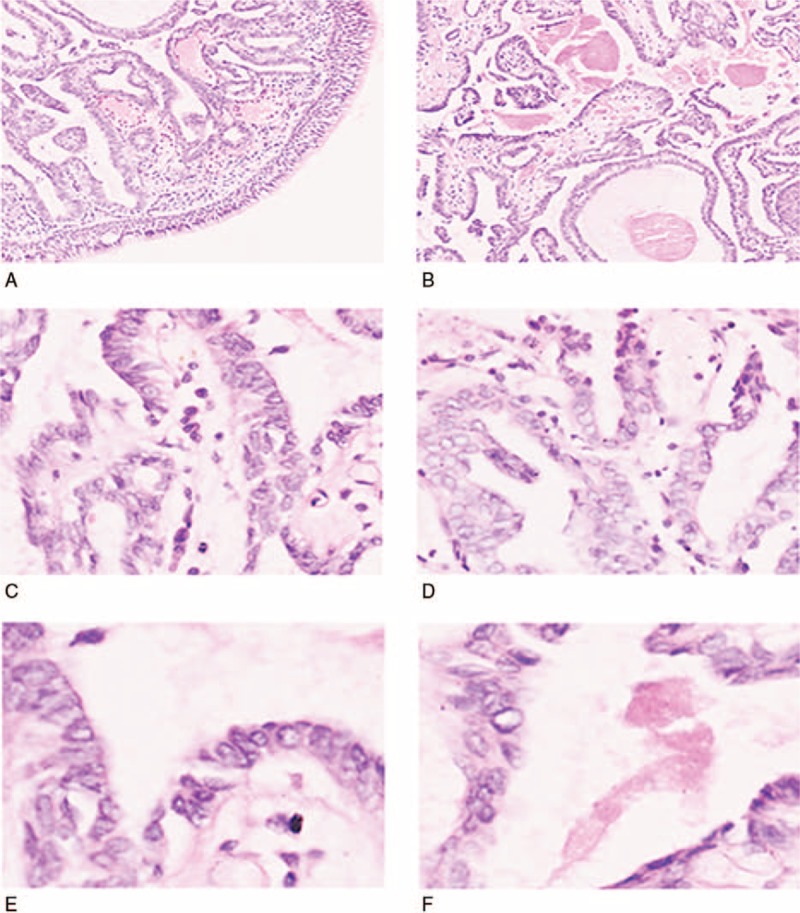
Histopathological features of TL-LGNPPA. (A) Primary thyroid-like nasopharyngeal papillary adenocarcinoma showing invasive growth pattern (100×). (B) Papillary architecture seen in TL-LGNPPA (100×). (C) Papillae with central fibrovascular core (400×). (D) Tumor cell nuclei with ovoid shape, ground-glass appearance, and occasional nuclear grooves (400×). (E) Tumor cells in a crowded arrangement with ground-glass nuclei and occasional nuclear grooves (630×). (F) Tumor cells with thickened nuclear membrane and irregular nuclear shape (630×).

Histopathological examination of the biopsy and surgical specimen demonstrated tumor cells within the nasopharyngeal subepithelium with an invasive growth pattern (Fig. 1A). The tumor cells were arranged in papillary structures with fibrovascular cores (Fig. 1C). The tumor cells were predominately arranged in a monolayer but showed focal crowding (Fig. 1B, D–F). The cell nuclei were predominately ovoid with irregular nuclear contours, a thickened nuclear membrane, and a ground-glass appearance. Nuclear grooves were also focally identified (Fig. 1E, F). After careful examination by 3 pathologists, no obvious pathological mitotic figures, necrosis, lymphovascular, or perineural invasion was identified (Fig. 1).

**Figure 2:**
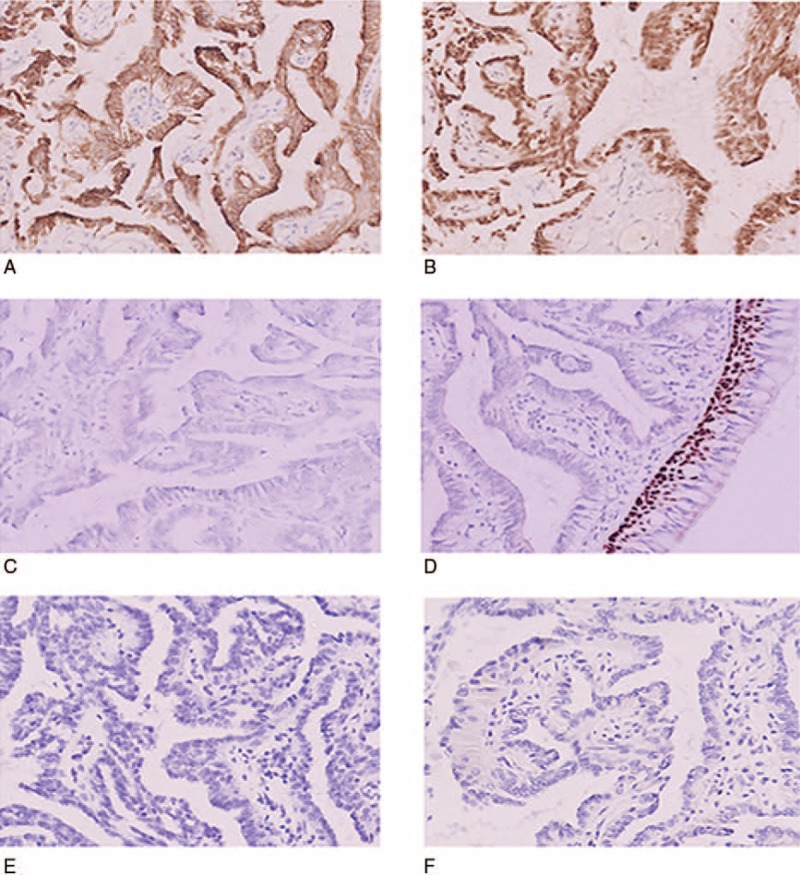
Immunohistochemical features of TL-LGNPPA. (A) Strong positive staining for CK7 (IHC staining, 200×). (B) Positive nuclear staining for TTF-1 (IHC staining, 200×). (C) Negative expression of TG (IHC staining, 200×). (D) Negative expression of P63 in tumor cells with positive control in basal cells (IHC staining, 200×). (E) Negative in situ hybridization investigation for the presence of small EBV-encoded RNA (EBER) (200×). (F) Negative high-risk HPV in situ hybridization (200×).

Immunohistochemical studies were performed. The tumor cells demonstrated positive expression for CK7 (Fig. 2A), CK19, thyroid transcription factor-1 (TTF-1; Fig. 2B), and CEA. The tumor cells were negative for thyroglobulin (TG; Fig. 2C), S-100, P63 (Fig. 2D), CK5/6, CK20, prostate-specific antigen (PSA), CDX-2, napsin A, and PAX-8. As for the association between nasopharyngeal carcinoma and Epstein–Barr Virus (EBV), in situ hybridization investigation for small EBV-encoded RNA (EBER) was negative (Fig. 2E). High- and low-risk human papilloma virus (HPV) in situ hybridization was also performed and was negative (Fig. 2F). Follow-up continued for 12 months after the surgery at which time there were no signs of recurrence and no evidence of metastatic disease observed by laboratory testing and imaging studies.

## 3 Discussion

The histological features of LGNPPA and TL-LGNPPA are similar to each other; however, TL-LGNPPA is an extremely rare neoplasm morphologically resembling papillary thyroid carcinoma with aberrant TTF-1 expression.^[1]^ Based on previous 15 English reports,^[1–3,6–15]^ TL-LGNPPAs showed a pedunculated or polypoid mass, ranging in size from <1 up to 3 cm (median 2 cm). The lesion is most commonly located in the superior and posterior aspects of the nasopharynx and arises from the surface epithelium. The age of patients affected ranged from 9 to 68 years old with a median age of 34 years. There is no predilection for male versus female. There is currently no standard treatment available for this disease. Effective treatment for TL-LGNPPAs has included complete surgical resection, but if surgical resection is not ideal, radiotherapy can be used as an adjuvant treatment.^[5,16]^ In addition, no cases of recurrence have been reported, indicating excellent prognosis.

It is difficult to morphologically distinguish TL-LGNPPAs from primary nasopharyngeal low-grade papillary adenocarcinomas, salivary gland-type polymorphous low-grade adenocarcinomas, and metastatic papillary thyroid carcinoma.^[3,4]^ The tumor cells of these lesions may all have a papillary architecture with an invasive growth pattern and slight nuclear atypia. However, immunohistochemical analysis shows different characteristics. We summarized the characteristic IHC expression of reported TL-LGNPPAs (Table 1). TL-LGNPPAs are characterized by the positive expression of TTF-1, whereas salivary gland-type polymorphous low-grade adenocarcinomas show positive expression of S-100.^[4,9]^ According to the previous study, LGNPPAs are negative for TTF-1 or S-100 expression.^[4,5,9,17]^ It is also noted that TL-LGNPPAs are similar to metastatic papillary thyroid carcinomas where both demonstrate papillary architecture, ground-glass nuclei, and nuclear grooves. Interestingly, metastatic papillary thyroid carcinomas are always positive for TG, but only one reported case of TL-LGNPPA showed focal expression of TG.^[13]^ Meanwhile, clinical manifestations, imaging results, and more comprehensive immunohistochemical staining could also exclude the possibility of metastatic papillary thyroid carcinoma as well as other metastatic carcinomas. In addition, negative expression of PSA, CDX-2, and napsin A are helpful to exclude metastatic prostate cancer, intestinal-type adenocarcinomas, and lung adenocarcinoma, respectively.^[18–20]^

**Table 1:**
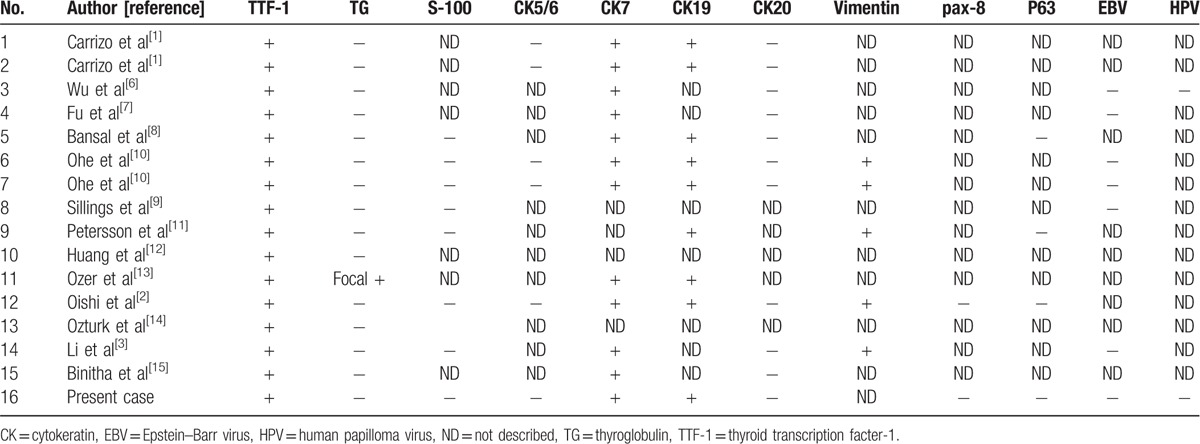
Summary of immunohistochemistry of reported primary thyroid-like low-grade nasopharyngeal papillary adenocarcinoma.

To the best of our knowledge, TTF-1 is a homeodomain-containing transcription factor coded by NKX2-1 in the development of the epithelial cells of the lung and thyroid. Therefore, the specific expression of TTF-1 is commonly used in the diagnosis of lung and thyroid cancers. In recent reports, the positive expression of TTF-1 was also observed in other carcinomas of the breast, colon, ovary, endometrium, and bladder, both in the primary tumor and metastatic sites, as well as neuroendocrine tumors.^[21]^ However, the mechanism of abnormal TTF-1 expression in TL-LGNPPAs is unclear and further studies should be performed.

The majority of malignant nasopharyngeal neoplasms are closely associated with EBV infection, but in situ hybridization for EBV was negative in our case and also in previously reported cases. Although HPV positivity is exceedingly rare in nasopharyngeal carcinomas, HPV high- and low-risk testing was still performed and negative results were also observed.^[6,22]^ Because of the limited number of cases, the relationship between TL-LGNPPAs and EBV or HPV needs to be further studied.

## 4 Conclusions

In conclusion, TL-LGNPPA is an extremely rare malignant nasopharyngeal tumor with papillary architecture and positive expression of TTF-1. When the pathologist receives a primary mass from the nasopharynx with such features, TL-LGNPPA should be on the differential diagnosis. Therefore, we propose this rare case with detailed clinical and histopathological features in order to attract more attention to this diagnosis and help pathologists and clinicians make appropriate diagnostic and therapeutic decisions.
